# Tissue specific imprinting on innate lymphoid cells during homeostasis and disease process revealed by integrative inference of single-cell transcriptomics

**DOI:** 10.3389/fimmu.2023.1127413

**Published:** 2023-03-07

**Authors:** Peng Song, Ke Cao, Yonghuan Mao, Shichao Ai, Feng Sun, Qiongyuan Hu, Song Liu, Meng Wang, Xiaofeng Lu, Wenxian Guan, Xiaofei Shen

**Affiliations:** ^1^ Department of Gastrointestinal Surgery, Nanjing Drum Tower Hospital, The Affiliated Hospital of Nanjing University Medical School, Nanjing, China; ^2^ Department of Gastrointestinal Surgery, Nanjing Drum Tower Hospital, Drum Tower Clinical Medical College of Nanjing Medical University, Nanjing, China; ^3^ Department of Critical Care Medicine, Nanjing Drum Tower Hospital, The Affiliated Hospital of Nanjing University Medical School, Nanjing, China

**Keywords:** innate lymphoid cells, single-cell transcriptomics, tissue imprinting, cell heterogeneity, integrative inference

## Abstract

**Introduction:**

Innate lymphoid cells (ILCs) are key components of the immune system, yet the similarity and distinction of the properties across tissues under homeostasis, inflammation and tumor process remain elusive.

**Methods:**

Here we performed integrative inference of ILCs to reveal their transcriptional profiles and heterogeneity from single-cell genomics. We collected a large number of ILCs from human six different tissues which can represent unique immune niches (circulation, lymphoid tissue, normal and inflamed mucosa, tumor microenvironment), to systematically address the transcriptional imprinting.

**Results:**

ILCs are profoundly imprinted by their organ of residence, and tissue-specific distinctions are apparent under pathological conditions. In the hepatocellular carcinoma microenvironment, we identified intermediate c-kit^+^ ILC2 population, and lin^-^CD127^-^ NK-like cells that expressed markers of cytotoxicity including *CCL5* and *IFNG*. Additionally, CD127^+^CD94^+^ ILC1s were preferentially enriched in inflamed ileum from patients with Crohn’s disease.

**Discussion:**

These analyses depicted a comprehensive characterization of ILC anatomical distribution and subset heterogeneity, and provided a base line for future temporal or spatial studies focused on tissue-specific ILC-mediated immunity.

## Introduction

Innate lymphoid cells (ILCs) are newly discovered lymphocytes which are functionally analogous to polarized CD4^+^ T helper (Th) cells, acting as important contributors to the regulation of immunity, inflammation, and tissue homeostasis (1). ILCs lack antigen-specific receptors on their surface, making them distinct from dendritic cells (DCs), macrophages, B and T lymphocytes (termed Lineage negative, Lin^-^) (1, 2). ILCs have the capacity to adjust to tissue-specific environments and respond to shocks or infections by producing cytokines, which direct and enhance immune responses on the front line of attack (2–4). Currently, ILCs are well characterized into five categories: NK cells, lymphoid-tissue inducer (LTi) cells together with group 1, 2 and 3 ILCs (ILC1s, ILC2s and ILC3s, respectively). ILC1s (CD127^+^CD117^−^CRTH2^−^) and NK cells (CD127^−^) share several common features, including their expression of the transcription factor T-bet (encoded by *TBX21*), the responsiveness to IL-15, IL-12 and IL-18, and the capacity to secrete interferon-γ (IFN-γ). Most NK cells are also dependent on EOMES for lineage specification, and it appears the characteristics of killer cells in immunosurveillance, whereas ILC1s are innate helper cells with weaker cytotoxic activity (5–7). With regard to ILC2s (CD127^+^CD117 ^±^ CRTH2^+^), GATA3 is the master transcriptional regulator for their development, as well as their capacity of producing IL-5 and IL-13 that govern a wide spectrum of features, including type 2 immune response, helminth infection and tissue fibrosis (8). ILC3s (CD127^+^CD117^+^CRTH2^−^) are defined by RORγt (encoded by *RORC*) expression, produce IL-17and IL-22, which have important functions in lymphoid tissue development and protection against extracellular bacteria and fungi (9, 10).

Recently, the single-cell RNA sequencing (scRNA-seq) technologies have been increasingly applied to characterize tissue-specific imprinting of ILCs, which improved our knowledge of the human immune system’s diversity. For example, Mazzurana et al. identified a novel unconventional ILC2 subpopulation (CRTH2^–^ ILC2) in the lung, expressing receptors for IL-33 and IL-25, by full-length Smart-seq2 scRNA-seq (11). Heinrich et al. discovered c-Kit^+^ ILC2s increased in frequency from non-tumor liver to tumor tissue in contrast to NKp44^−^ ILC3s (12). Among the ILC3s, Rethacker et al. identified a CD56^+^ILC3 subset expressed cytotoxicity genes (such as *PRF1*, *GZMA* and *GZMB*) shared with NK cells, which infiltrated metastatic breast cancer lymph nodes (13). Besides, combination of measurements of many cellular properties by flow cytometry and unbiased cell type identification by scRNA-seq allowed us to find the best cellular parameters to purify any cell type of interest (14).

In this study, using a combination of published scRNA-seq data, we painted the landscapes of ILCs across six human tissues including blood, colon, lung, tonsil, inflamed ileum from Crohn’s disease patients and hepatocellular carcinoma (HCC). By characterizing the degree of commonalities and differences of those subsets in different tissues, we aim to reveal the diversity and plasticity of human ILCs, and extend understanding of innate immune system.

## Methods

### Single-cell RNA-seq datasets collected in this study

We searched PubMed databases through September 2022 using the following search terms: single-cell RNA sequencing OR scRNA-seq AND ILC. In order to find any relevant studies, the manual search was augmented by carefully reading the reference lists from those retrieved publications. For inclusion, the study must meet the following criteria: (1) human tissue samples were obtained from adult or pediatric donors (excluding fetal tissues); (2) for the scRNA-seq characterization, ILCs were purified by flow cytometry (CD45^+^ Lineage^-^); (3) raw sequencing reads or count matrix could be obtained. When study populations were overlapped or duplicated in some studies, we chose the most complete and suitable research.

### Single-cell RNA-seq data processing and integration

For collected scRNA-seq datasets, cell annotation tables (ILC types) were obtained from the original publications; otherwise, we applied Seurat (version 4) with default parameters to filter and identify ILC types. Briefly, cells with fewer than 200 genes detected or > 40% mitochondrial counts or > 50% ribosomal counts were filtered out; genes detected in > 3 cells and UMI count > 1000 were kept. The GSE179795 dataset was run *via* Scrublet to eliminate any potential doublets, setting the expected doublet rate to 0.05 (15).

Considering the heterogeneity from different platforms and different studies, we applied “computesumFactors” function from scran R package to compute the size factor of each cell, which in turn was used to normalized the counts (16). A log2 transformation was also performed on normalized counts. Next, we used the “modelGeneVar” function to compute the biological variation for each gene, and the top 2000 genes were identified as highly variable features (HVGs). Mitochondrion, ribosomal and cell cycle genes were not interesting in our research, and thus were excluded in HVGs. Then gene expression matrix was scaled to z-score which could remove much of the batch effect caused by differences in platforms. To further handle more batches, “harmony” (17) or “LIGER” (18) was applied immediately after PCA. For visualization, the dimensionality of this combined dataset was further reduced using Uniform Manifold Approximation and Projection (UMAP).

In order to quantitatively evaluate the performance on integration, the Local Inverse Simpson Index (LISI) that defines the effective number of datasets in a neighborhood of a cell was calculated (17). LISI represents the local neighborhood of a sample with respect to the batch (integration LISI, iLISI) or accuracy (cell-type LISI, cLISI), respectively. While in good and accurate integration, higher iLISI value indicated that neighborhoods presented by more datasets and lower batch effect; lower cLISI value reflected a separation of unique cell types throughout the embedding and thus accurate integration.

### Tissue distribution of clusters

To quantify the tissue preference of ILC subsets, odds ratios (OR) were calculated. As Zheng et al. described, the OR is a disproportional measure based on the ratio of the odds of one ILC subset in a specifical tissue, compared to the odds of remaining ILC subsets in the other tissues (19). One subset was identified as being enriched in a specific tissue if OR > 1.5.

### Differential expression analysis

To identify differentially expressed genes for cluster demarcation, “FindAllMarkers” function in Seurat was used. GO enrichment analysis was performed using the clusterProfiler R package separately for differentially upregulated genes (adjusted p-value < 0.05) for each cluster (20). The functional gene sets belonging to biological process were focused and selected with an adjusted p-value cutoff of 0.05. Cytokines were annotated according to the “KEGG_CYTOKINE_CYTOKINE_RECEPTOR_INTERACTION” gene set. Only the cytokine genes that were detected in at least 0.25 percent of cells in either of the two populations were retained.

### Pseudo-bulk differential expression analysis across different tissues

Given that our samples are from different tissues, it was crucial to consider sample-to-sample variation. Therefore, pseudo-bulk samples were created for differential expression testing based on the subpopulation-stratified scRNA-Seq data (21, 22). Specifically, we summed counts transcript per gene for each subpopulation per sample, and then performed DESeq2 differential analysis (21). Pseudo-bulk samples consisting of fewer than 10 cells were removed.

## Results

### Integration of multiple scRNA-seq datasets

**Figure 1:**
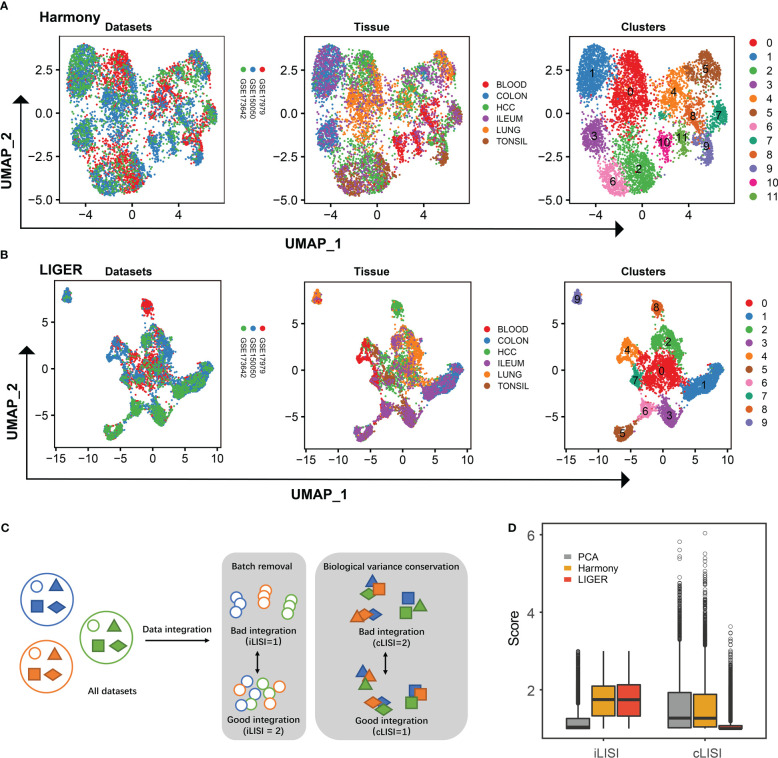
Integration of ILCs from three scRNA-seq datasets. **(A)** UMAP plots showing the integration of three scRNA-seq datasets by Harmony. **(B)** UMAP plots showing the integration of three scRNA-seq datasets by LIGER. **(C)** Schematic workflow to the quantitative assessment of data integration and batch-correction fidelity. iLISI metrics and cLISI provide measures of the degree of mixing among datasets and integration accuracy on cell types, respectively. **(D)** Boxplot showing the iLISI and cLISI distribution of data integrated by PCA, Harmony and LIGER.

We compiled an integration of single-cell transcriptomics of ILC across six human tissues (**Supplementary Table 1**). As Mazzurana et al. described, one minor cluster of cells lacking expression of *PTPRC* (encoding CD45) and another minor cluster of cells coordinating antigen-presenting function (e.g., high expression of *HLA-DQA1*, *HLA-DRA* and *HLA-DPA1*) were identified and removed (11). After stringent quality-control filtering, we obtained 6670 ILCs from 20 samples, derived from three single-cell RNA-seq datasets [GSE150050 (11), GSE179795 (12) and GSE173642 (23)]. As shown in **Figures 1A, B**, we used Harmony and LIGER to integrate the above three datasets, respectively. Both Harmony and LIGER successfully removed batch effects from different platforms, whereas Harmony exhibited low bio-conservation score (**Figures 1C, D**). Thus, we used LIGER to integrate the above three scRNA-seq datasets for further analysis.

### Single-cell transcriptional landscape of ILCs in different tissues

**Figure 2:**
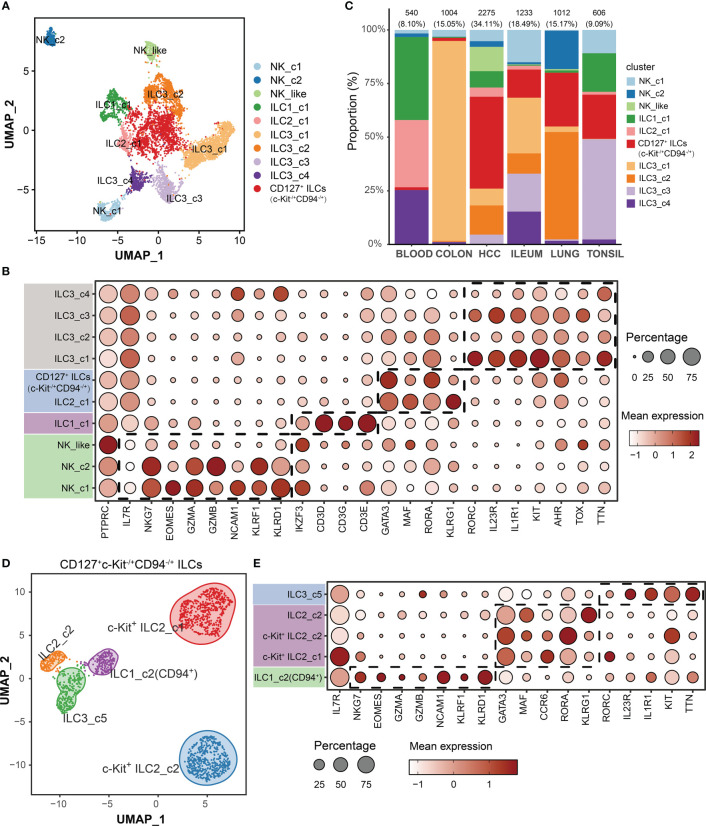
Single-cell transcriptome profiles of ILCs among different tissues. **(A)** UMAP visualization of all cells (6670) color-coded on the basis of annotated clusters. **(B)** Dot plot displaying average and percent expression of marker genes across ten ILC clusters. **(C)** Proportion of each cell type among different tissues. **(D)** UMAP visualization of c-Kit^-/+^ ILC2/ILC3 subset (1537 cells). **(E)** Dot plot displaying average and percent expression of marker genes for c-Kit^-/+^ ILC2/ILC3 subset across five clusters.

To characterize the subsets of 6670 ILCs among different tissues (blood, colon, lung, tonsil, ileum and HCC), we performed unsupervised graph-based clustering and then identified 10 clusters based on canonical cell markers (**Figures 2A, B**). Two clusters expressed *NKG7*, *EOMES*, *GZMA/B*, *NCAM1* (CD56), *KLRF1* and *KLRD1* (CD94) but lacking *IL7R* were annotated as NK cells (NK_c1 and NK_c2). One cluster lacked expression of *IL7R*, *NCAM1* and *KLRD1*, but expressed IL1 related genes (*IKZF3*), which shared characteristics of both NK cells and ILC1s, and thus named “NK_like” subset (12). One cluster was identified as ILC1_c1 subset, with higher levels of expression of specific transcription factor (*IKZF3*), and T cell markers (*CD3D*, *CD3G* and *CD3E*). One ILC2 subset (ILC2_c1) shared common ILC2s markers (*GATA3*, *MAF* and *RORA*). The ILC2-specialized gene *PTGDR2* (encoding CRTH2) was not examined because it was absent in GSE173642 dataset. Four clusters were characterized as ILC3s (ILC3_c1, ILC3_c2, ILC3_c3 and ILC3_c4) on the basis of uniquely high *RORC*, *IL23R*, *IL1R1*, *KIT*, *AHR*, *TOX* and *TTN* expression. We discovered c-Kit^+^ ILC2 specific clusters in the GSE179795 dataset and CD127^+^CD94^+^ ILC1 subset in GSE173642 dataset as a result of independent analysis of the included datasets (**Supplementary Figure 1**). Therefore, one cluster in this integrated graph with both *GATA3* and *KIT* high expressing, was annotated as CD127^+^c-Kit^-/+^CD94^-/+^ ILC subset for further analysis. The proportion of cells in these lineages varied highly among different tissues, indicating a heterogeneous cellular condition (**Figure 2C**). To further dissect in variability within the CD127^+^c-Kit^-/+^CD94^-/+^ ILC subset, 1537 aligned cells were re-clustered. Intriguingly, we found 5 clusters: one CD94^+^ ILC1 subset (ILC1_c2), two c-Kit^+^ ILC2 subsets, one c-Kit^-^ ILC2 subset (ILC2_c2) and one ILC3 subset (ILC3_c5) using above cell markers (**Figures 2D, E**; **Supplementary Figure 2**). Interestingly, ILC1_c2 subset consisted primarily of CD127^+^CD94^+^ ILCs from inflamed ileum in GSE173642 dataset (**Supplementary Figure 2**). Moreover, Krabbendam et al. reported that no IL-22 expression was detected when IL-23, IL-1, or IL-15 were used to cultivate with ex vivo isolated CD127^+^CD94^+^ ILCs; whereas IFN-γ expression was clearly detected in response to IL-15 or IL-12+IL-1β stimulation, which confirming that CD127^+^CD94^+^ cells belong to the group 1 ILCs (23). We also assigned each cluster to a cell type using gating strategy base on gene expression, which validated the clustering strategy is considerably accurate (**Supplementary Figure 3**).

### Transcriptome analysis of ILC1s

**Figure 3:**
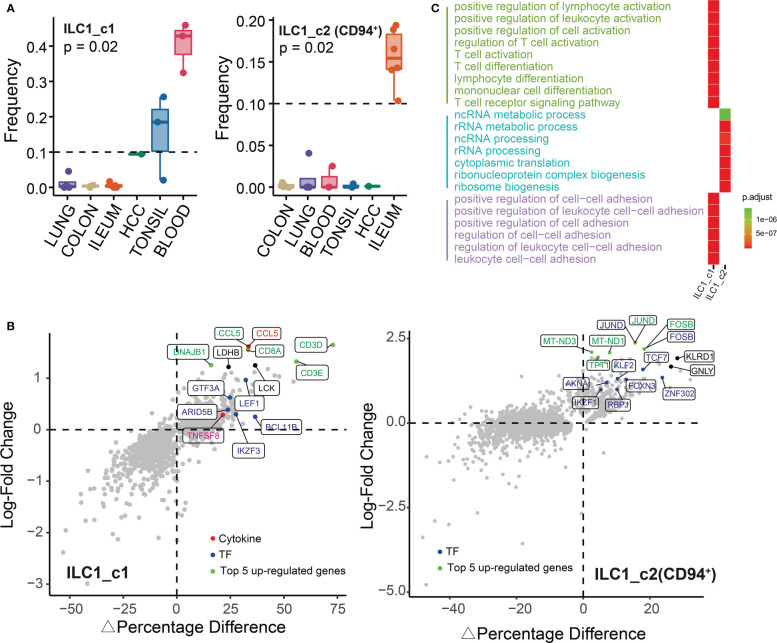
Characterization of ILC1s in different tissues. **(A)** Box plots showing the frequencies of ILC1s (divided by the total CD127^+^ ILC number) in lung, colon, ileum, tonsil, blood and HCC. P value were calculated from Kruskal-Wallis test. **(B)** The percentage of differentially expressed transcripts (△Percent of Cells and log-fold change) in ILC1s is shown. **(C)** Gene ontology analysis (biological processes) of differentially expressed genes showing top 15 GO terms in each ILC1 subset.

As shown in **Figure 3A**, we identified two ILC1 subsets: ILC1_c1 and ILC1_c2 (CD94^+^). ILC1_c1 population were mainly distributed in blood and tonsil. To identify unique profiles to the ILC1 populations, we compared the gene expression of ILC1s with the combined expression profile of genes in the other populations pooled together. Notably, ILC1_c1 cells had significantly higher expression of *CD3D*, *CD3E*, CD3G and *CD8A*, as previously described (11, 24), which were involved in T-cell development and signal transduction. Among these markers, *CD3D* was most strongly transcribed. This subset also expressed TCR-associated signaling molecules such as *LCK*, which has previously been regarded as T-cell specific gene, and glycolysis-associated transcript *LDHB* (**Figure 3B**). Furthermore, ILC1_c1 cells displayed differential expression of genes encoding transcription factors (*LEF1*, *BCL11B*, *IKZF3*, *GTF3A* and *ARID5B*) and secreted effectors (*TNFSF8* and *CCL5*). ILC1_c2 populations (CD127^+^CD94^+^ ILC1) co-expressed *IL7R*, *KLRD1*, *GNLY*, and shared characteristics of both NK cells and conventional ILCs. Theses ILC1s accumulated within inflamed ileum, and were proposed to contribute to inflammation. The ILC1_c2 subset also specifically expressed genes encoding *JUND* and *FOSB* (**Figure 3B**; **Supplementary Table 2**), belonging to functional component of the AP1 transcription factor complex, which appeared to have distinct effects on cell proliferation and transformation (25). By performing GO analysis, we noted the upregulation of functions associated with regulation of T cell activation and cellular homeostasis related to focal/cell adhesion. Besides, the ILC1_c2 subset of genes were enriched for cytoplasmic translation and ribosome biogenesis. (**Figure 3C**; **Supplementary Table 3**).

### Transcriptome analysis of ILC2s

**Figure 4:**
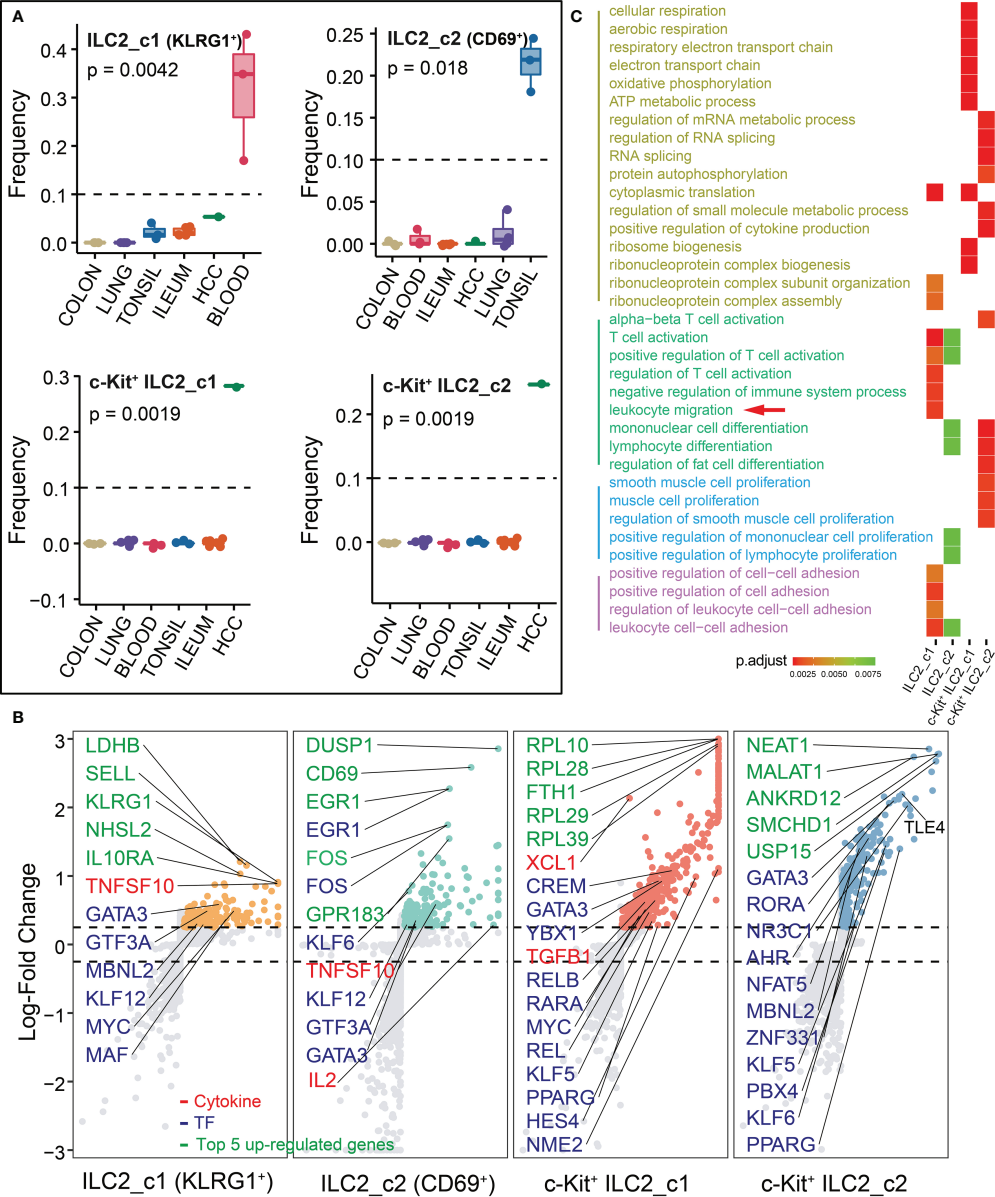
Characterization of ILC2s in different tissues. **(A)** Box plots showing the frequencies of four ILC2 subsets (divided by the total CD127^+^ ILC number) in lung, colon, ileum, tonsil, blood and HCC. P values were calculated from Kruskal-Wallis test. **(B)** Differential gene expression analysis showing up- and down-regulated transcripts across four ILC2 subsets. **(C)** Functional enrichment analysis showing top 15 GO terms in each ILC2 subset of biological process.

The distribution of ILC2s exhibited obvious cross-group differences, indicating surprising levels of heterogeneity among cells. We totally identified four subsets of ILC2s, and ILC2_c1 (KLRG1^+^) and ILC2_c2 (CD69^+^) were major ILC populations in the blood and tonsil, respectively. The other two subsets (c-Kit^+^ ILC2_c1 and c-Kit^+^ ILC2_c2) were only detected in HCC (**Figure 4A**). All subsets had high expression of transcription factor *GATA3* required for ILC2 development (**Figure 4B**; **Supplementary Table 2**). The TNF family-related transcripts *TNFSF10* (encoding TRAIL, also known as APO-2 ligand) were also highly expressed by ILC2_c1 and ILC2_c2 subsets, acting as a cytotoxic protein which activates rapid apoptosis in tumor cells, but not in normal cells (26). In addition, early differentiation markers, including *SELL*, *IL10RA*, and *KLRG1*, were expressed differentially in ILC2_c1 subset, that appeared to be in an immune quiescence state (27). Interestingly, circulating KLRG1^+^ ILC2s from individuals with allergic disease were unable to generate IL-10, but this ability was recovered after effective allergen immunotherapy (8, 28). CD200R1 encoding a receptor for the OX-2 membrane glycoprotein was also shown to be expressed most highly on ILC2_c1 subset (**Supplementary Table 2**). The tissue-residency marker CD69 was mostly expressed in ILC2_c2 subset. Within the ILC2_c2 population, several endogenous regulators of the anti-inflammatory response (*DUSP1* and *EGR1*) were identified (28). Specifically, one study analyzed the nasal epithelium exposed to grass-pollen allergen, and found IL-10-producing ILC2s reduced the expression of pro-inflammatory genes *NFKB1* and *MYC* in an IL-10-dependent manner and increased the expression of the anti-inflammatory transcription factor *EGR1* (28).

A strong enrichment of ribosomal protein encoding genes (starting with RPL- or RPS-) and metabolism-related gene (*FTH1*) in c-Kit^+^ ILC2_c1 subset, indicating a higher level of oxidative phosphorylation and metabolic changes which was similar with our previous work (29). Among the genes encoding secreted proteins, the c-Kit^+^ ILC2_c1 population expressed *XCL1* that involved in the recruitment of dendritic cells (30), and fibrosis-associated gene *TGFB1* with either tumor-suppressing or tumor-promoting effects (31, 32). The c-Kit^+^ ILC2_c2 population highly expressed *TLE4*, which belongs to Groucho family members function as transcription co-repressors within the context of Wnt signaling (33). We next performed GO analysis on all populations, leading us to note that the pathways mainly involved in T cell activation (typical associated genes: *LEF1*, *PAG1*, *TESPA1*, *B2M*, *IL2*; ILC2_c1 and c2 subsets), leukocyte migration (*S1PR1*, *SELL*, *CD200R1*, *ANXA1*, *SELPLG*; ILC2_c1 subset), metabolic process (cytoplasmic translation, *RPS26*, *RPL36A*, *RPL10*; ATP metabolic process, *ALDOA*, *CHCHD10*, *TMSB4X*; c-Kit^+^ ILC2_c1 subset), and regulation of RNA splicing (*AHNAK*, *FUS*, *RBM39*, *PIK3R*; c-Kit^+^ ILC2_c2 subset; **Figure 4C**; **Supplementary Table 3**).

### Transcriptome analysis of ILC3s

**Figure 5:**
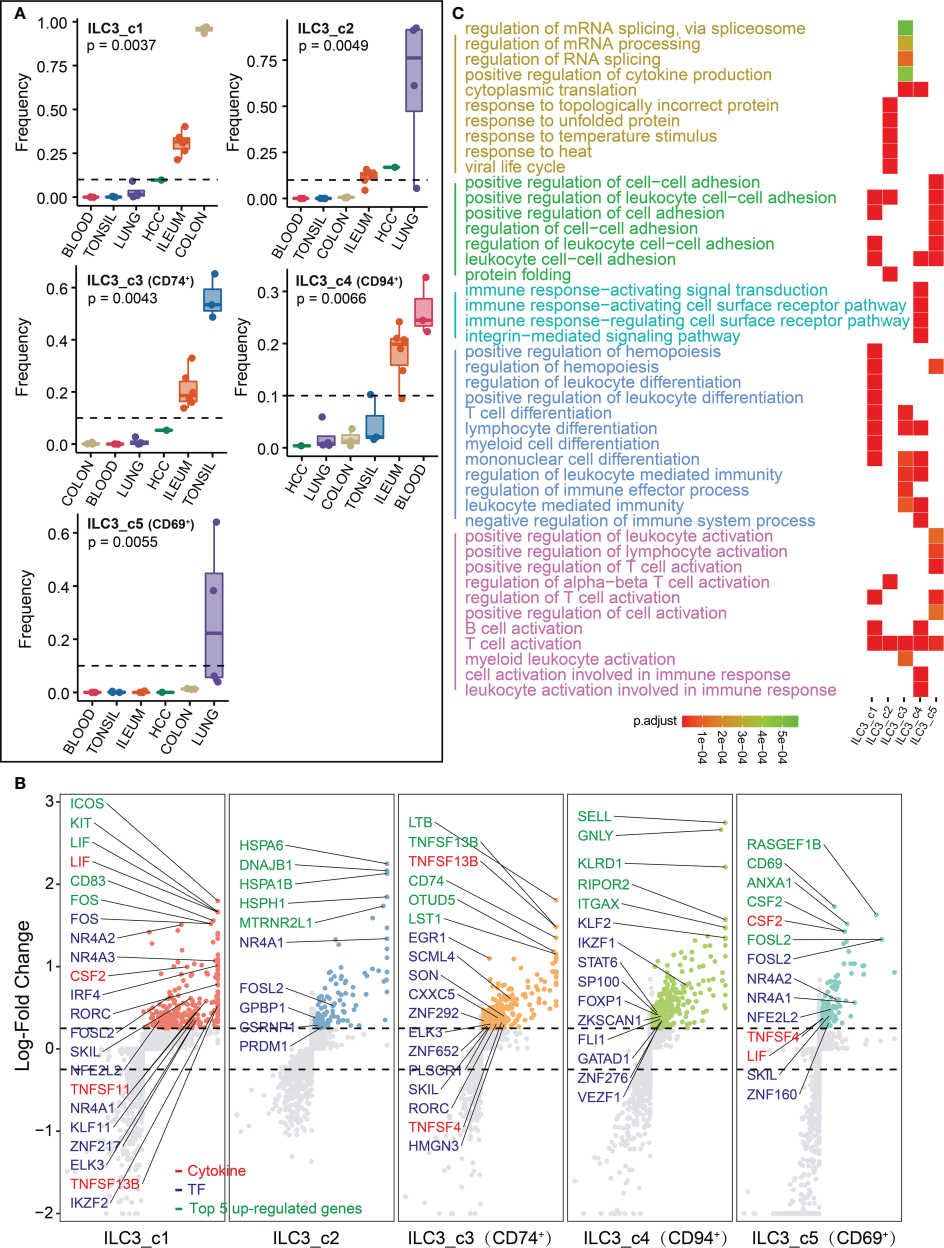
Characterization of ILC3s in different tissues. **(A)** Box plots showing the frequencies of four ILC3 subsets (divided by the total CD127^+^ ILC number) in lung, colon, ileum, tonsil, blood and HCC. P values were calculated from Kruskal-Wallis test. **(B)** Differential gene expression analysis showing up- and down-regulated transcripts across six ILC3 subsets. **(C)** Functional enrichment analysis showing top 15 GO terms in each ILC3 subset of biological process.

Each tissue has its unique ILC3 subsets distribution. For instance, in the colon ILC3_c1 subset is the most predominant; the lung is populated by ILC3_c2 and ILC3_c5 (CD69^+^) subsets; ILC3_c3 (CD74^+^) subset is most relevant in tonsil; while ILC3_c4 (CD94^+^) are widely localized in blood and ileum, respectively (**Figure 5A**). To further reveal the features of these ILC3 clusters, we next examined the top five highly expressed genes, transcriptional factors, cytokines, respectively (**Figure 5B**; **Supplementary Table 2**). The ILC3_c1 populations expressed high levels of transcription factors including Fos gene family (*FOS*, *FOSB* and *FOSL2*), steroid-thyroid hormone-retinoid receptor superfamily (*NR4A1*, *NR4A2* and *NR4A3*), indicating that Fos/Jun signaling and calcineurin/NFAT might be important for this subset differentiation (34, 35). In addition to the cytokines, this subset also expressed *LIF*, *CSF2*, *TNFSF11* and *TNFSF13B*, resulting in strong intestinal inflammation. In all this subset shared a common transcriptomic signature characteristic of typical ILC3s. The ILC3_c2 subset was enriched for heat shock proteins (*HSPA6*, *HSPA1B*, *HSPH1* and *DNAJB1*), suggesting that the unfolded protein response best distinguishes this population. The ILC3_c3 subset showed abundant amounts of *LTB* and *CD74* which have been demonstrated to have *in vivo* significance in murine models (36, 37), and *OTUD5* that also been shown to regulate RORγt stability (38). Nagasawa et al. reported that NKp46^+^ ILCs and tonsillar ILC3 populations shared the expression of CD74 that associated with antigen presentation for immune response, suggesting it as a distinctive marker for ILC3 (39). *LST1* was specifically upregulated in this subcluster, which can inhibit the proliferation of lymphocytes (**Supplementary Table 2**). The ILC3_c4 subset was transcriptionally less active, and could be characterized by expression of *SELL* and *ITGAX*, involved in cell adhesion and migration, and *KLRD1* with *GNLY*, expressed in T and NK cells, might correspond to cytotoxic ILC3s. The residency marker CD69 was strongly expressed in ILC3_c5 subset. Strikingly, consistent with ILC3_c1 subset, the high expression of proliferation genes *NR4A1*, *NR4A2* and *FOSL2*, cytokine genes *CSF2* and *LIF* were also highly expressed in the ILC3_c5 subset, further indicating colon and lung might share associated transcriptional imprinting of ILC3. ILC3-derived CSF2 controlled macrophages and dendritic cells to maintain colonic Treg homeostasis (40). Besides, high expression of *TNFSF4* (encoding OX40 ligand) was observed in ILC3_c3 and c5 subsets. Deng et al. revealed the importance of OX40-OX40L signaling for intestinal homeostasis by the crosstalk between Tregs (OX40) and ILC3s (OX40L) (41). Consistently, we found that the enriched GO terms were in line with the function of aforementioned genes (such as regulation of immune effector processes and responses, **Figure 5C**; **Supplementary Table 3**).

### Transcriptome analysis of NK and NK_like cells

**Figure 6:**
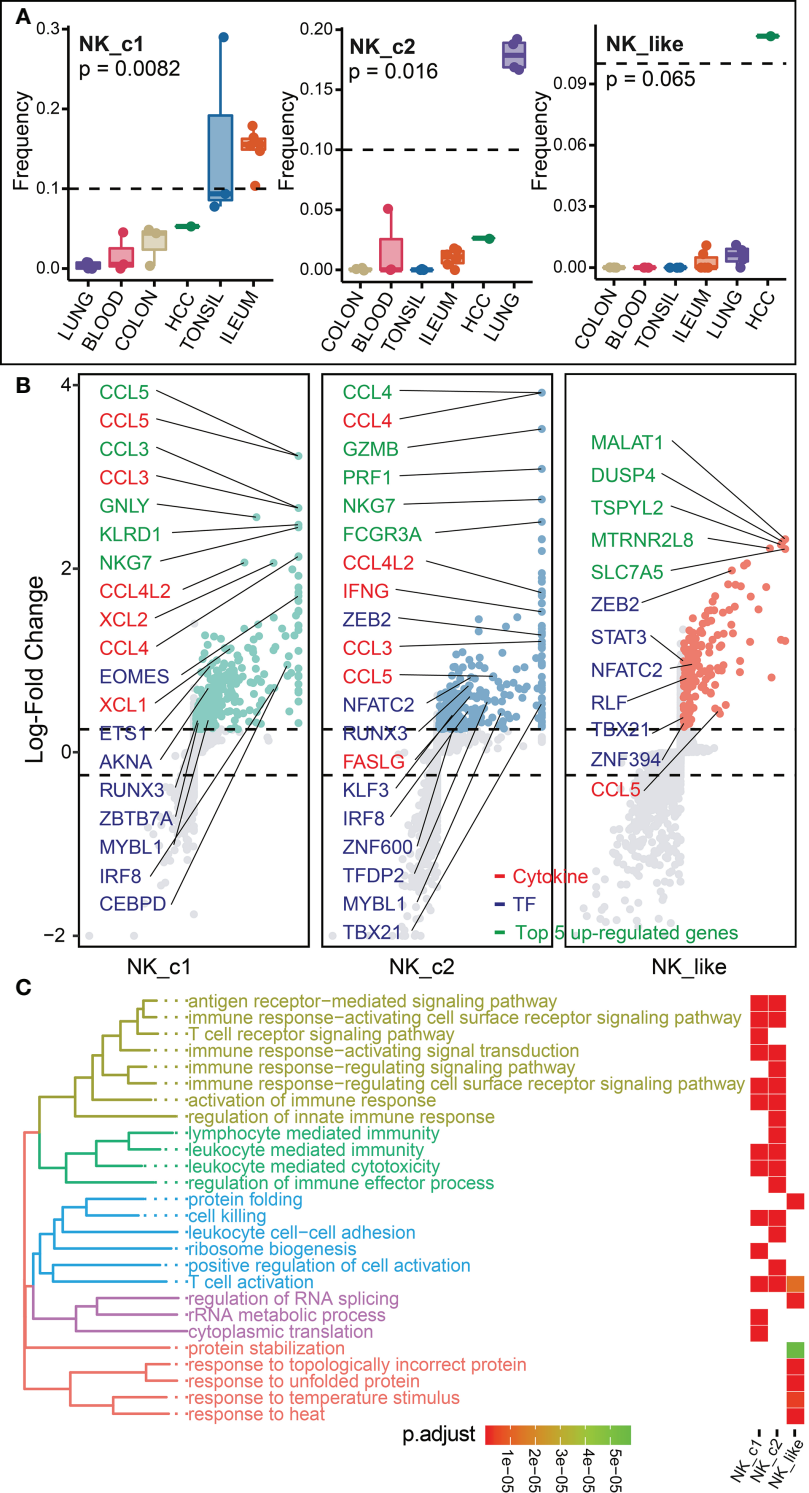
Characterization of NK and NK_like cells in different tissues. **(A)** Box plots showing the frequencies of four NK subsets (divided by the total ILC number) in lung, colon, ileum, tonsil, blood and HCC. P values were calculated from Kruskal-Wallis test. **(B)** Differential gene expression analysis showing up- and down-regulated transcripts across three NK subsets. **(C)** Functional enrichment analysis showing top 15 GO terms in each NK subset of biological process.

We next explored the transcriptome changes for NK and NK_like cells, and found that differentiated NK cells predominated in tonsil, ileum (NK_c1) and lung (NK_c2), and NK_like cells distributed in HCC (**Figure 6A**). Differential expression analysis showed that NK cell subsets shared relatively high expression of cytokines (*CCL3*, *CCL4*, *CCL5*, *CCL4L2*, *FASLG* and *IFNG*, **Figure 6B**; **Supplementary Table 2**). The NK_c1 population highly expressed *EOMES* that are related T-box transcription factors that control NK cell development (7). The NK_c2 and NK_like population uniquely expressed *ZEB2* encoding a transcriptional regulator involved in terminal NK cell maturation (42). Biological process enrichment analysis demonstrated that the transcriptome of NK and NK_like cells was specifically enriched in cell cytotoxicity and immune response (**Figure 6C**; **Supplementary Table 3**).

### Characteristics and pseudotemporal analysis of ILCs

**Figure 7:**
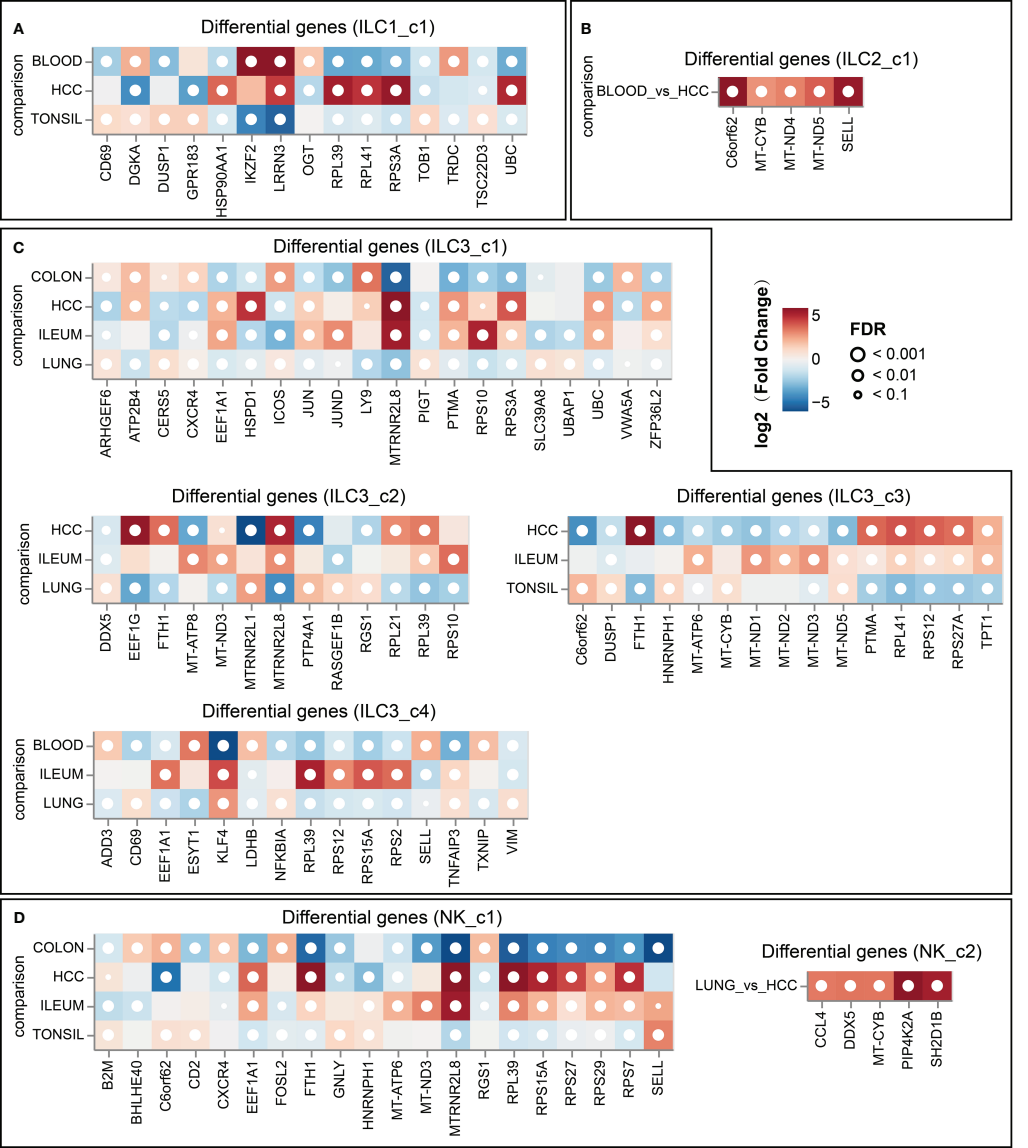
Top 5 differentially expressed genes in ILC subsets (**A**, for ILC1_c1 subset; **B**, for ILC2_c1 subset; **C**, for ILC3 subsets; **D**, for NK subsets) across different tissues (DESeq2 on pseudo-bulk).

**Figure 8:**
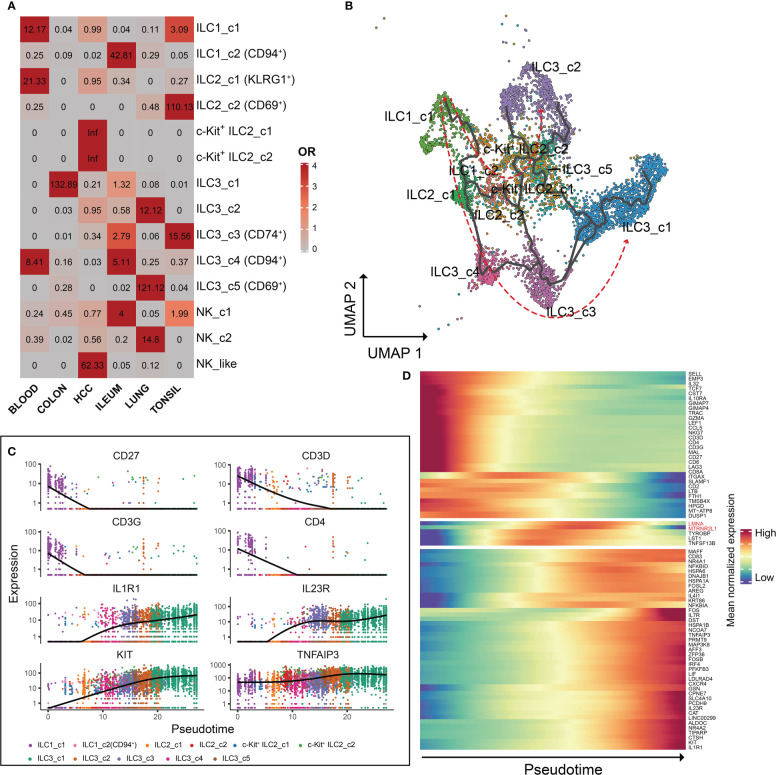
Characteristics and pseudotemporal gene expression analysis of the ILCs across different tissues. **(A)** Tissue prevalence of ILC clusters estimated by OR values. OR > 1.5 indicates that this ILC cluster is preferred to distribute in the corresponding tissue. **(B)** Pseudotime inference of the ILCs (excluding NK cells) trajectory with cells color-coded according to the corresponding ILC subsets identified by UMAP. **(C)** Monocle analysis reveals the kinetics of indicated genes (*CD27*, *CD3D*, *CD3G*, *CD4*, *IL1R1*, *IL23R*, *KIT* and *TNFAIP3*) across pseudotime. **(D)** Heatmap of gene expression analysis associated with ILC specification in Monocle.

To explore the profile of ILC subsets across different tissues, we continued the analysis of pseudo-bulk samples. As shown in **Figure 7**, the specificity of ILC distribution was also observed in pseudobulk data (**Supplementary Table 4**). ILC1_c2, ILC2_c2, c-Kit^+^ ILC2_c1/2, ILC3_c5 and NK-like subsets were the predominant ILC populations in the corresponding tissues, since the number of related ILCs within the other tissues was less than 10. Several encoding transcription factors (*KLF4*, *FOS*, *JUN* and *JUND*) and early activation markers (*CD69* and *DUSP1*) were unregulated in unique tissues or different disease state (43). We further used OR values to quantified tissue enrichment of ILC subsets across different tissues (**Figure 8A**). The compositions of ILCs from different tissues displayed prominent differences, especially to c-Kit^+^ ILC2 groups that appeared to be tumor-enriched. The proximity in UMAP coordinates of ILCs (excluding NK and NK_like cells) indicated that their transcriptomes might be connected continuously. Next, we searched for a potential developmental path by applying the pseudotime algorithm. As shown in **Figure 8B**, the trajectory evinced a pseudotemporal order of ILCs, and placed individual ILCs into different states with striking fidelity to our annotations. The root of the trajectory was mainly populated by ILC1s, and the two termini of the tree were populated mainly by ILC3_c1 and ILC3_c2. This pseudotemporal analysis further helped identify genes associated ILC1s from the root to both fates such as *CD27*, *CD3D*, *CD3G*, and also genes related with ILC3s that were up-regulated in cells differentiating into both fates such as *IL1R1*, *IL23R* and *KIT* (**Figure 8C**). Additionally, ILC2-associated genes *LMNA* (highly expressed in c-Kit^+^ ILC2_c1 subset), *MTRNR2L12* (highly expressed in c-Kit^+^ ILC2_c2 subset) were mostly expressed in the intermediate state, indicating the plasticity of ILC2s (44) (**Figure 8D**).

## Discussion

Recent studies of ILCs have focused on their subsets’ specific roles in these processes like tissue remodeling and repair, immune-mediated disease, lymphoid tissue production, intestinal and mucosal defense, and epithelial homeostasis (2, 9). We collected a large number of ILCs from human six different tissues which can represent unique immune niches (circulation, lymphoid tissue, normal and inflamed mucosa, tumor microenvironment), to systematically address the transcriptional imprinting. Through this approach, we have identified tissue-specific ILC subsets with the potential to remodel the local environment.

Two subpopulations of ILC1 cells were characterized in our study. ILC1_subset showed a strong distribution preference in blood and tonsil, and exhibited high levels of *IKZF3*, followed the regulation pattern of T-bet and EOMES, being predominantly expressed by ILC1 and NK cells (45). ILC1_c2 subset (CD94^+^) exhibited distinct phenotypic profile, tended to be enriched in inflamed ileum, and highly expressed *GNLY*, encoding for the cytotoxic protein granulysin that is linked to chemotaxis or fugetaxis of monocytes and bacterial lysis. While exploring the heterogeneity of NK cells, we identified two “conventional” NK subsets (CD127^-^CD56^+^CD94^+^), and a NK-like subset (CD127^-^CD56^-^CD94^-^IKZF3^+^) that expressed markers of cytotoxicity including *IFNG* and *CCL5*. The CD127^-^NK_like cell population mainly presented in the HCC microenvironment, which was in accord with the results Heinrich et al. described (12).

ILC2s concentrate at barrier surfaces, such as the skin, lung and intestine, where they are phenotypically and functionally adapted to the tissue specific environment (46). Recent discoveries have generated profound insights into ILCs biology of circulating between different organs upon activation, as they are readily detectable in healthy human peripheral blood (8, 47). Notably, the ILC2_c1 cells identified within multiple tissues were preferentially enriched in blood, resembling the reported migratory ILC2s. These ILC2s increased expression of genes involved in cell migration such as *SELL*, *S1PR1* and *CD200R1*. Although distinct subsets of ILCs can be identified in blood, the precursor ILC (ILCp), which has the ability to home to peripheral tissues and differentiate into mature ILC subsets, makes up the majority of the circulating ILC population (48). For example, Winkler et al. observed that ILC2s accumulate in bronchoalveolar lavage fluid after allergen challenge in asthmatic patients, whereas circulating ILC2 numbers decrease at the same time, suggesting ILC2s are recruited from the blood into the lung during inflammation (49). Liu et al. have identified two migratory colon ILC2 subsets (IL-17A^+^ ILC2s and CD27^+^ ILC2s) with potential to migrate to the mouse lung in response to IL-25 stimulation (35). As mentioned above, *SELL* is also highly expressed in ILC3_c4 subset, which was preferentially enriched in blood and inflamed ileum. Krabbendam et al. revealed that co-expression of *SELL* and *CCR7* in CD127^+^CD94^+^ granulysin-expressing ILCs are capable of traveling to the lymph node *via* high endothelial venules (23). Additionally, we observed two unique c-Kit^+^ ILC2 subsets in HCC, reflecting plasticity as induced by the tumor environment. Heinrich et al. reported that supernatant derived from HCC tissue with high IL-6 and TGF-β induced an increase in c-Kit^+^ ILC2s. In parallel, the frequency of NKp44^-^ ILC3s and ILC1s was reduced (12). Bernink et al. similarly observed that TGF-β regulates the transition of c-Kit^-^ ILC2s into IL-17 producing c-Kit^+^ ILC2 cells, and this switch was reliant on RORγt and the downregulation of GATA3 (50).

Analysis of human tissue samples shows that diversity spectrum of ILC3s is dependent on the local environment. ILC3s were previously further subdivided according to the expression of CD56 and especially of NKp44. Human NKp44^+^ ILC3s are the most prevalent cells in adult tonsil and gut, and represent an exclusive source of IL-22 (51). We also observed *NCR2* (NKp44) highly expressed in ILC3_c1 and ILC3_c3 subsets that were enriched in colon and tonsil, respectively (**Supplementary Figure 4**). We further found these two subsets also expressed *ENTPD1* (CD39), an ectoenzyme related to suppression of inflammation (**Supplementary Figure 4**). Strictly, CD39 was mainly restricted to NKp44^+^ ILC3 cells in mucosal tissues (52). ILC3_c1 subset is the predominant source of intestinal ILC-derived LIF, which belongs to IL-6 family cytokines, and restricts inflammation by blocking Th17-cell differentiation (53). ILC3s (except ILC3_c2 subset) demonstrated high levels of histocompatibility complex class II (MHCII) associated transcripts including *HLA-DQB1*, *HLA-E*, *HLA-F* and *HLA-DRA*. Goc et al. reported that a dialog between ILC3s and CD4 T cells *via* major MHCII is disrupted in colon cancer, resulting in destruction of immunologic homeostasis in the gut and tumor microenvironment (54). Interestingly, we also found ILC3_c4 subset abundantly expressed high levels of *GNLY* and *KLRD1*, that mediate cellular cytotoxicity. ILC3s can be harnessed for cytotoxic responses *via* differentiation with IL-12 and IL-15 stimulation as described previously (55, 56). In healthy gut mucosa, Qi et al. similarly found a population of ILC3/NK cells that had transcriptomic features in common with both ILC3 and NK cells (24). This population expressed *NKG7*, *KLRD1*, *GNLY*, *XCL2* and *CCL4* similar to NK cells, but differed from conventional ILC3s. However, Björklund et al. has described a subset with high *SELL* expressing as naive ILC3 cells in tonsil (57). CD62L^+^ (*SELL*) ILC3s didn’t respond to IL-23 plus IL-1β stimulation, and neither IL-22 nor IL-17F was detected, but produced IL-2 relatively infrequently. The biological role of this ILC3 subset remained to be addressed. Using scRNA-seq technology, clusters in the UMAP plot expressed helper ILC-related genes (*IL7R* and *KIT*), but lacked transcripts that encode for effector functions, were usually annotated as naïve ILCs (nILCs) (11, 23). In ILC-poiesis, nILCs may represent the functional equivalent of ILC precursors. In our joint analysis, we did not discover this cluster, which might be caused by the simultaneous integration across donors and technologies. Regarding the activation state, ILC3_c5 (CD69^+^) subset was preferentially enriched in lung and expressed high levels of *CSF2* (GM-CSF), which plays an important role in antimicrobial pulmonary host defense function and is essential for surfactant homeostasis (58).

The past decade has witnessed an explosion of knowledge of innate immune cell, owing to the definition of many additional subsets. In this study, we found several ILC subsets across six tissues, that exhibited tissue-adapted phenotypes and functions. The typical ILC clusters with specific markers could be distinguished easily, whereas some cells had transcriptomic features in common, rendering their identification complicated. Therefore, we defined a population of CD127^+^c-Kit^-/+^CD94^-/+^ cells, and further divided it into five clusters, indicating the plasticity between ILC subsets, which can be induced by changes in the tissue microenvironment. However, we defined theses subsets based an unbiased transcriptome-wide perspective, *ex vivo* co-culture experiments were not performed to confirm their functions.

Overall, the main objective of this study was to characterize the heterogeneity of ILCs in human tissues under physiological and pathological conditions. Integrative inference of these single-cell transcriptomics unveiled the diversity and plasticity of ILCs, and generated a comprehensive map of human ILC composition, which provided insights into ILC-mediated tissue-specific immunity.

## Data availability statement

Three single-cell RNA-seq datasets (GSE150050, GSE179795 and GSE173642) used in this study can be obtained from the GEO database (https://www.ncbi.nlm.nih.gov/geo/). The original contributions presented in the study are included in the article. Further inquiries can be directed to the corresponding authors.

## Author contributions

Conception and design: PS, XS and WG. Collection and assembly of data: PS, KC, YM and XL. Data analysis and interpretation: PS, XS, KC, YM, SA, FS, QH, SL and MW. Manuscript writing: PS. Manuscript revision: KC, YM, XS and WG. All authors read and approved the final manuscript.

## Supplementary material

Supplementary Figure 1 Single-cell RNA sequencing of HCC for GSE179795 dataset. **(A)** UMAP of single-cell transcriptomes color-coded by ILC clusters. **(B)** Dot plot displaying average and percent expression of marker genes across seven ILC clusters.

Supplementary Figure 2 Single-cell transcriptome profiles of CD127^+^c-Kit^-/+^CD94^-/+^ ILC subset among different tissues. **(A)** Heatmap of top 3 signature genes for indicated cell types were shown. **(B)** Expression of *KLRD1* (CD94) plotted in purple on the UMAP clustering. **(C)** Proportion of each indicated cell type among different tissues.

Supplementary Figure 3 Gating strategy to identify ILCs using multi-omics single-cell sequencing data. Representative gates were generated to assign ILCs (ILC1, ILC2, ILC3 and NK+NK_like cells) based on identified specific population markers.

Supplementary Figure 4 Single-cell transcriptome profiles of ILC3 subset among different tissues. **(A)**: Dot plot displaying average and percent expression of representative genes across five ILC3 clusters. **(B)** Expression of *NCR2* (NKp44) and *ENTPD1* (CD39) in purple on the UMAP clustering.
