# Supplementary figures and images for: Tissue specific imprinting on innate lymphoid cells during homeostasis and disease process revealed by integrative inference of single-cell transcriptomics
Source: Front Immunol. 2023 Mar 7;14:1127413. doi: 10.3389/fimmu.2023.1127413 (PMC10028295; doi:10.3389/fimmu.2023.1127413)

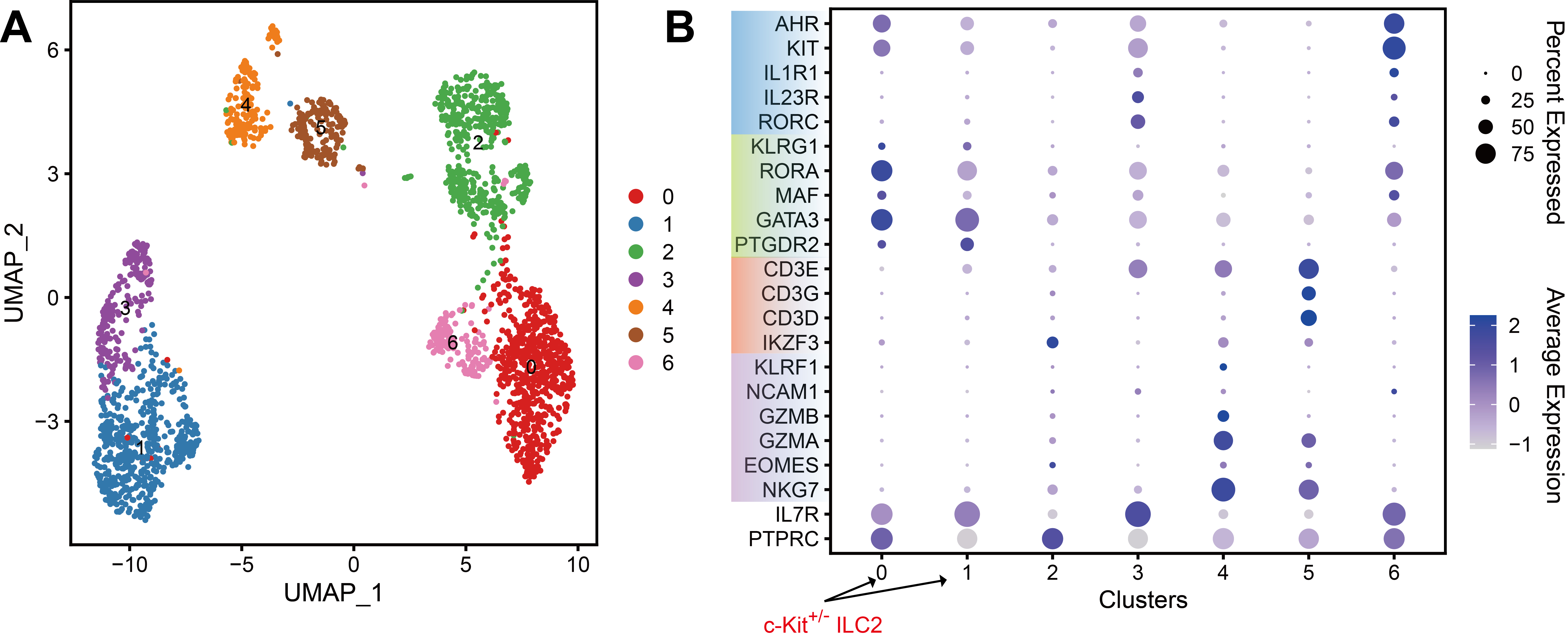

Supplement: Supplementary Figure 1 — Single-cell RNA sequencing of HCC for GSE179795 dataset. (A) UMAP of single-cell transcriptomes color-coded by ILC clusters. (B) Dot plot displaying average and percent expression of marker genes across seven ILC clusters. [file Image_1.jpeg]

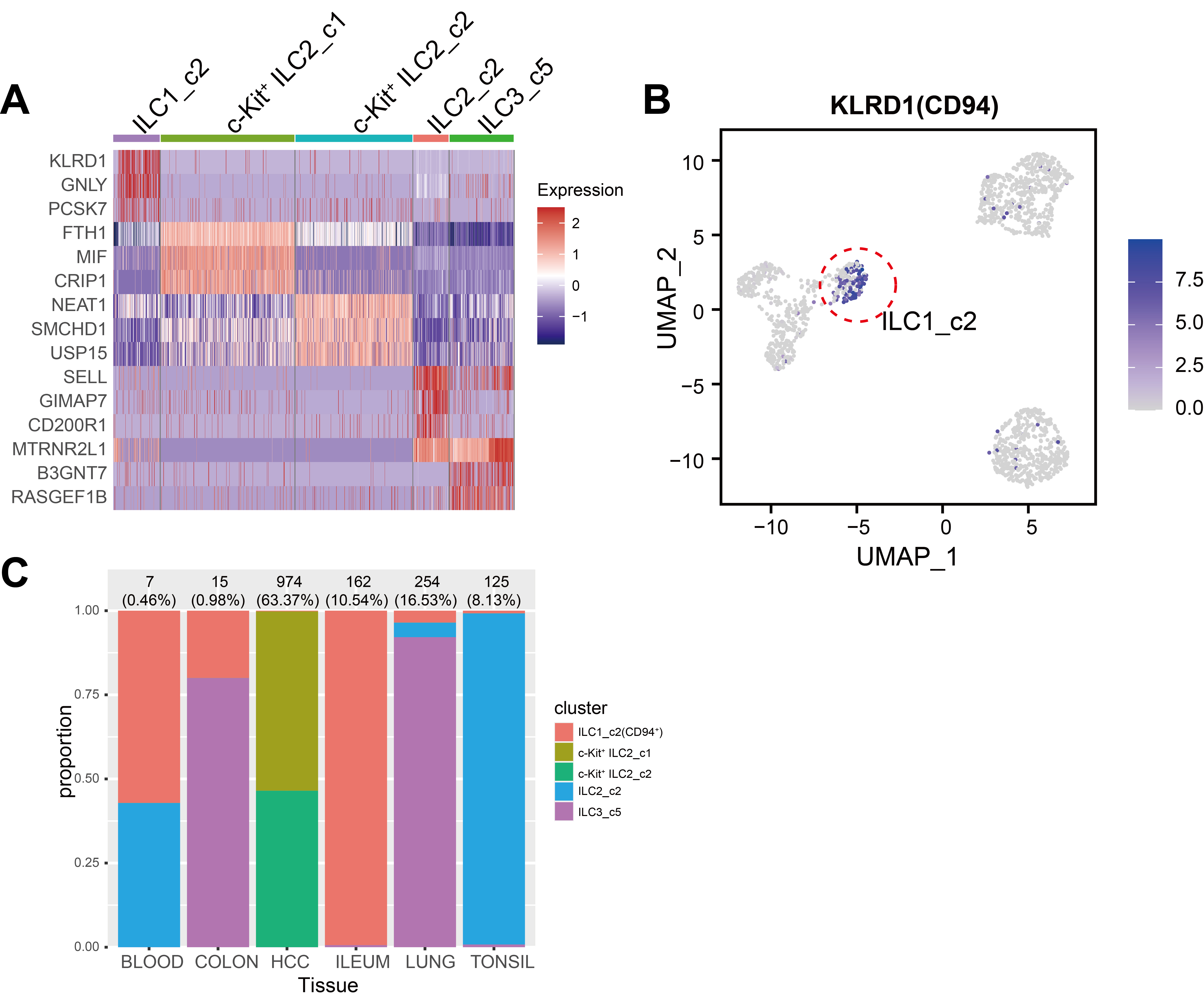

Supplement: Supplementary Figure 2 — Single-cell transcriptome profiles of CD127+c-Kit-/+CD94-/+ ILC subset among different tissues. (A) Heatmap of top 3 signature genes for indicated cell types were shown. (B) Expression of KLRD1 (CD94) plotted in purple on the UMAP clustering. (C) Proportion of each indicated cell type among different tissues. [file Image_2.jpeg]

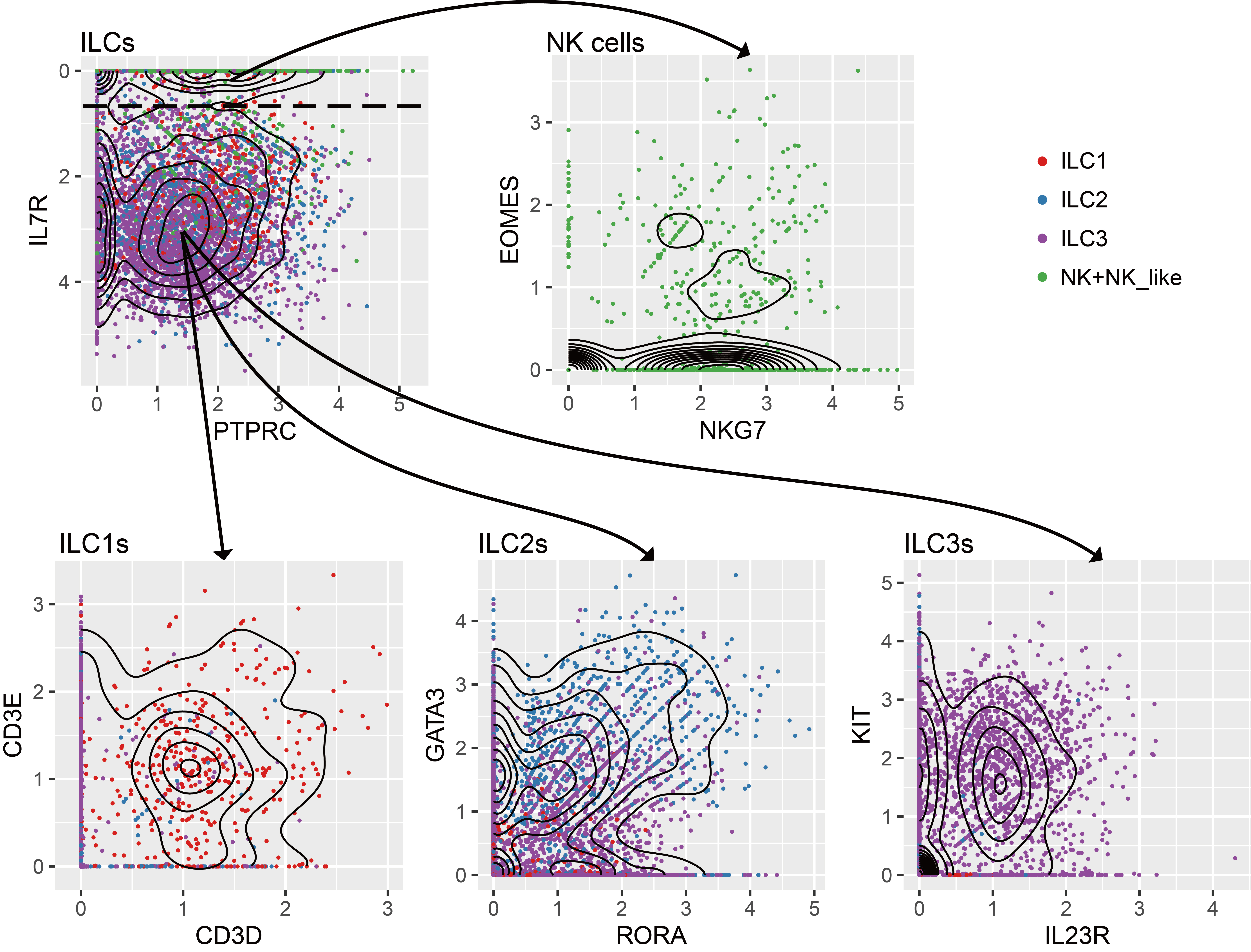

Supplement: Supplementary Figure 3 — Gating strategy to identify ILCs using multi-omics single-cell sequencing data. Representative gates were generated to assign ILCs (ILC1, ILC2, ILC3 and NK+NK_like cells) based on identified specific population markers. [file Image_3.jpeg]

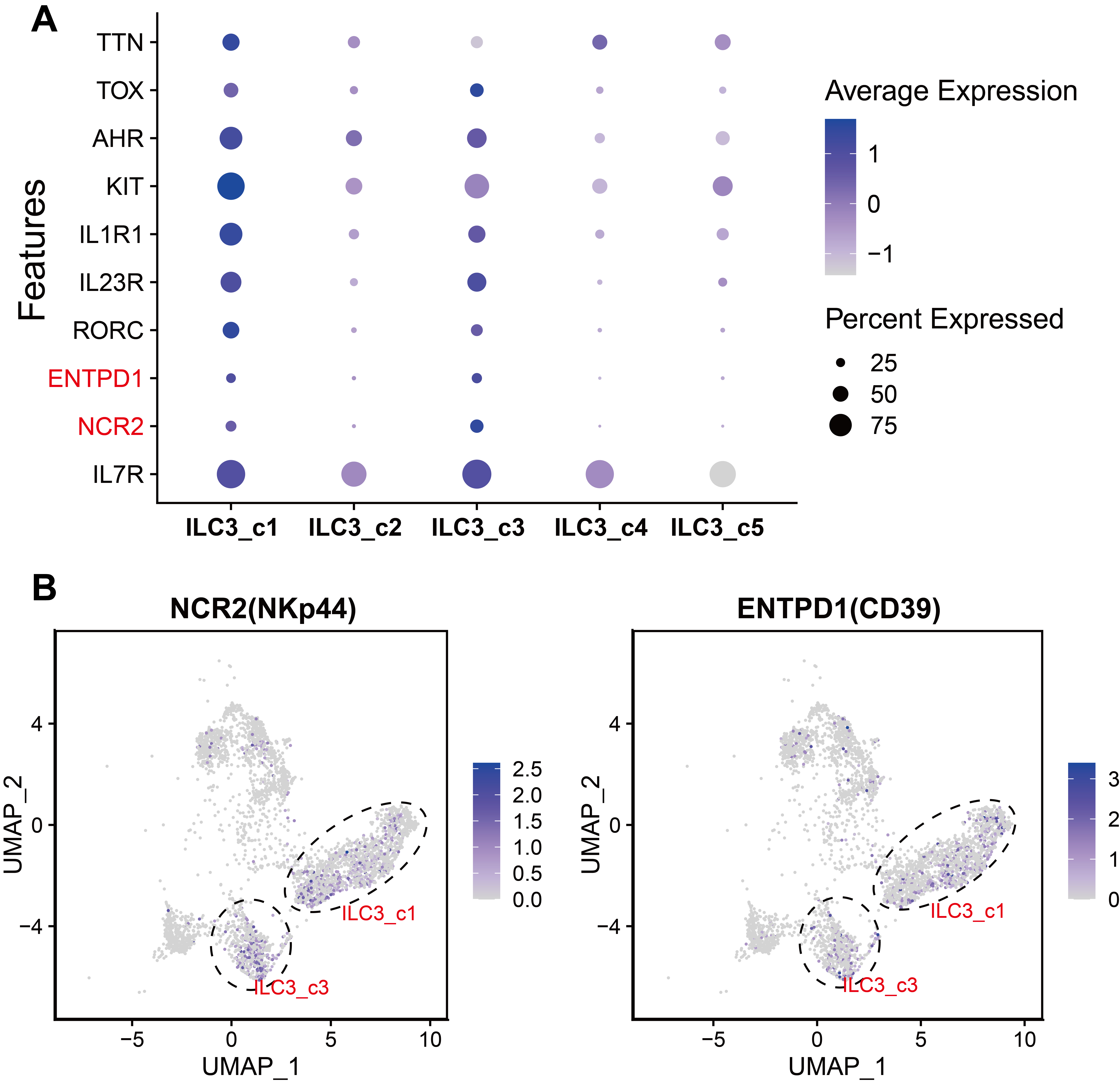

Supplement: Supplementary Figure 4 — Single-cell transcriptome profiles of ILC3 subset among different tissues. (A): Dot plot displaying average and percent expression of representative genes across five ILC3 clusters. (B) Expression of NCR2 (NKp44) and ENTPD1 (CD39) in purple on the UMAP clustering. [file Image_4.jpeg]
