# Supplementary material for: Tissue specific imprinting on innate lymphoid cells during homeostasis and disease process revealed by integrative inference of single-cell transcriptomics
Source: Front Immunol. 2023 Mar 7;14:1127413. doi: 10.3389/fimmu.2023.1127413 (PMC10028295; doi:10.3389/fimmu.2023.1127413)
Supplement: Supplementary file 5 [file Table_1.docx]

| **Author** | **Year** | **Tissue** | **No. of sample** | **ILC types** | **Platform** | **Dataset** |
| --- | --- | --- | --- | --- | --- | --- |
| Mazzurana *et al*.^a^ | 2021 | Lung | 4 | CD127^+^ ILCs  CD56^dim^CD16^+^ NK cells | Smart-seq2 | GSE150050 |
| Mazzurana *et al*.^b^ | 2021 | Blood | 3 | CD3^-^CD127^+^ ILCs | Smart-seq2 | GSE150050 |
| Mazzurana *et al*.^b^ | 2021 | Colon | 3 | CD3^-^CD127^+^ ILCs  CD56^+^ NK cells | Smart-seq2 | GSE150050 |
| Björklund *et al*.^c^ | 2021 | Tonsil | 3 | CD127^+^ ILCs  CD56 ^bright^ NKG2A^+^NK cells | Smart-seq2 | GSE150050/GSE70580 |
| Heinrich *et al*.^d^ | 2021 | Hepatocellular carcinoma | 1 | CD127^+^CRTH2^-^c-Kit^-^NKp44^-^ ILC1s  CD127^+^CRTH2^+^c-Kit^-/+^ILC2s  CD127^+^CRTH2^-^c-Kit^+^NKp44^-/+^ ILC3s | 10x Genomics | GSE179795 |
| Krabbendam *et al*.^e^ | 2021 | Inflamed ileum | 6 | CD127^+^ ILCs  CD94^+^NK cells | CEL-Seq2 | GSE173642 |

**Supplementary Table1:** Characteristics of including studies in this integrative study.

Lineage (lin) cocktail contained the following antibodies:

^a^Lin: CD1a, CD14, CD19, CD3, CD34, CD94, CD123, BDCA2, FcεR1, TCRαβ, TCRγδ

^b^Lin: CD1a, CD14, CD19, CD34, CD94, CD123, BDCA2, FcεR1, TCRαβ, TCRγδ

^c^Lin: CD1a, CD14, CD19, CD3, CD34, CD123, BDCA2, FcεRIα, TCRα/β, TCRγ/δ

^d^Lin: CD1a, CD34, CD3, TCRα/β, TCRγ/δ, CD14, CD19, CD16, CD94, CD123, BDCA2, FCeR1a

^e^Lin: CD1a, CD3, CD4, CD5, CD14, CD19, CD34, CD123, CRTH2, BDCA2, TCRαβ, TCRγδ, and FcER1α
